# Burden of Antimicrobial Resistance: Compared to What?

**DOI:** 10.1093/epirev/mxab001

**Published:** 2021-03-12

**Authors:** Marlieke E A de Kraker, Marc Lipsitch

**Keywords:** causal inference, causality, global burden of disease, drug resistance, microbial, methods, research design

## Abstract

The increased focus on the public health burden of antimicrobial resistance (AMR) raises conceptual challenges, such as determining how much harm multidrug-resistant organisms do compared to what, or how to establish the burden*.* Here, we present a counterfactual framework and provide guidance to harmonize methodologies and optimize study quality. In AMR-burden studies, 2 counterfactual approaches have been applied: the harm of drug-resistant infections relative to the harm of the same drug-susceptible infections (the susceptible-infection counterfactual); and the total harm of drug-resistant infections relative to a situation where such infections were prevented (the no-infection counterfactual). We propose to use an intervention-based causal approach to determine the most appropriate counterfactual. We show that intervention scenarios, species of interest, and types of infections influence the choice of counterfactual. We recommend using purpose-designed cohort studies to apply this counterfactual framework, whereby the selection of cohorts (patients with drug-resistant, drug-susceptible infections, and those with no infection) should be based on matching on time to infection through exposure density sampling to avoid biased estimates. Application of survival methods is preferred, considering competing events. We conclude by advocating estimation of the burden of AMR by using the no-infection and susceptible-infection counterfactuals. The resulting numbers will provide policy-relevant information about the upper and lower bound of future interventions designed to control AMR. The counterfactuals should be applied in cohort studies, whereby selection of the unexposed cohorts should be based on exposure density sampling, applying methods avoiding time-dependent bias and confounding.

## Abbreviations

AMR: antimicrobial resistance
MDR: multidrug resistance
MSSA: methicillin-susceptible *Staphylococcus aureus*
MRSA: methicillin-resistant *Staphylococcus aureus*
R: resistant
S: susceptible

## INTRODUCTION

Increasing focus on the problem of antimicrobial resistance (AMR) creates a need to quantify its impact on human health to provide evidence to guide and prioritize mitigation efforts. Such efforts raise conceptual and technical challenges, the most fundamental of which is to define what we mean by the impact or burden or harm caused by AMR. Important aspects of the harm caused by AMR may be quantified as requiring a longer hospital stay for treatment or resulting in greater mortality rates or loss of disability-adjusted life-years due to treatment failure or occurrence of long-term sequelae. Whatever the measure of harm, the thornier question is: “compared to what?” In this article, we tackle this conceptual challenge, proposing a hypothetical intervention-based approach (1) to consider the burden of AMR. We argue that 2 definitions of burden are salient and that, ideally, researchers should attempt to estimate quantities on the basis of both of these definitions and then use them to evaluate the harm avertable by different types of interventions. We also address the technical challenges of how studies should be designed to estimate these quantities from data.

## DEFINING THE BURDEN

**Figure 1:**
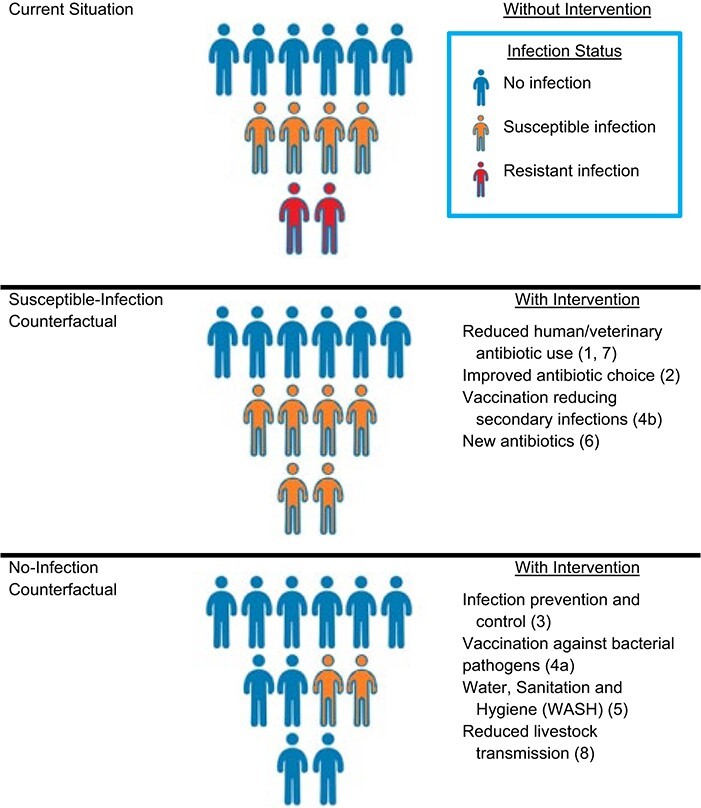
Possible counterfactual scenarios for specific interventions against drug-resistant infections. Numbers in parentheses refer to the numbered categories in the section titled “Which interventions might approximate which counterfactual?*”*.

Some have proposed that the precise question that burden-of-resistance studies should answer is, “How much harm is done by antibiotic-resistant infections, relative to the harm the same infections would do if they were susceptible to all antimicrobial drugs that are normally effective against that species of bacteria?” This question focuses on the harm resistant infections do *because* they are resistant. We refer to this question as estimating the impact of resistance compared to a susceptible (S)-infection counterfactual (Figure 1) (2).

Alternatively, some propose to estimate the burden of resistance by asking the question, “How much harm is done by antibiotic-resistant infections, relative to a situation in which such infections did not occur at all because they were prevented (e.g., by better infection control or a vaccine?)” This question focuses on the *total* harm resistant infections do. We refer to this question as estimating the impact of resistance compared to a no-infection counterfactual (Figure 1)*.*

Authors of a recent systematic review of 286 burden-of-resistance studies for World Health Organization priority bacteria found that 152 of them (53%) compared outcomes of resistant versus susceptible infections, 11 (4%) compared outcomes of resistant infections versus uninfected comparators, and 6 (2%) compared all 3 groups, with case-control studies more often making the comparison to uninfected patients (3). Notably, 89 burden-of-resistance studies did not include a comparison and reported crude health outcomes, or attributable health outcomes based on subjective chart review.

In other areas of epidemiology, it has been suggested that coherent causal questions can be asked only by positing a well-defined, specific intervention (feasible or hypothetical) (1) and designing a statistical analysis to mimic a randomized trial of that intervention, where the causal contrast of interest would be the contrast between those randomly assigned to receive the intervention and those randomly assigned to a control group (4). Here, we apply this perspective to the question of AMR burden. We begin by considering what might be the effect of various interventions to control AMR, and which (if either) of the 2 counterfactuals might be better approximated by the results of a successful intervention of various sorts. We conclude that some types of intervention might be expected to approximate 1 or the other of these counterfactuals, but we note there are several cases where the expected outcome is unclear. Therefore, we recommend that burden-of-AMR estimates consider both counterfactual comparisons.

### Which interventions might approximate which counterfactual?

Because AMR infections result from a complex set of causes whose influence and interactions remain poorly understood, the list of interventions that could potentially reduce its burden is long. Categories of interventions that should reduce the harm caused by resistant infections include: 1) reducing antimicrobial use, especially unnecessary use (e.g., through antimicrobial stewardship programs); 2) improving the choice of antimicrobial agent, dose, and therapy duration, including by the use of rapid resistance diagnostics, antimicrobial stewardship programs, or redacted reporting of susceptibility results by the microbiological laboratory (5, 6); 3) improving infection control aimed at reducing a) transmission of pathogenic bacteria in hospitals and other settings where antimicrobial use is high (7) and b) the probability of infection by a patient’s own flora (e.g., via improved procedures for catheter placement) (8); 4) introducing and promoting the use of vaccines (9) a) against infections that are commonly resistant and b) against infections that lead to substantial antibiotic use, exerting selection on bystander bacteria in persons who are treated (10); 5) improving water, sanitation, and hygiene—a form of infection control in the community (11); 6) developing and introducing new antibiotics (12); 7) reduction of environmental contamination or agricultural use of antibiotics; 8) livestock vaccination; and 9) combinations of these, such as increased use of resistance diagnostics to optimize therapy (category 2) combined with preferential efforts to reduce transmission from resistant cases (category 3) (13).

For each of these categories, we can consider their likely effects as either 1) reducing resistant infections without increasing susceptible ones, making the no-infection counterfactual more relevant; or 2) reducing the proportion of infections that are resistant while leading to some replacement of those infections with susceptible ones, making the S-infection counterfactual more relevant (Figure 1).

1. *Reductions in antimicrobial use.* We can think of this as an evolutionary intervention that reduces selection pressure for resistant strains. On the basis of this understanding, one might expect that reducing antimicrobial use would lead to an evolutionary shift away from resistant infections in favor of their susceptible competitors. We discuss later in this article the evidence for competition, or a lack thereof, between S and R strains of the same bacterial species. If such competitive interactions are assumed, mathematical models typically predict that reduced antibiotic use in the hospital will lead to reductions in the prevalence of R strains, with an offsetting increase in S strains (14). Alternatively, if resistant and susceptible strains do not compete with one another or do not compete very strongly, then reduced antimicrobial use may not lead to much or any compensatory increase in S infections as R infections decline. Despite these last caveats, the S-infection counterfactual seems most relevant for evaluating the possible outcomes of antimicrobial use reductions.
2. *Improving the choice of antibiotics*. This includes several different types of interventions, like cycling, or mixing at ward-level, combination therapy, alternating antibiotics (15), or using rapid diagnostics at the individual level. Cycling or mixing does not change the number of cases treated but only what they are treated with, likely resulting in a reduction of 1 resistant phenotype in favor of another resistant phenotype (16), in which case neither of the counterfactuals described here is precisely relevant. However, assuming that the individual-level interventions would increase the proportion of appropriately treated R infections, this would effectively convert them to S infections, indicating that the S-infection counterfactual could be relevant.
3. *Improving infection control in hospitals*. Intuitively, one might imagine that infection control (e.g., better hand hygiene and room sanitation in hospitals) would equally affect all strains of bacteria, R and S alike, and therefore, such a nonspecific intervention would reduce R and S infections equally. Mathematical models, however, suggest that if R and S strains compete with one another, and if R strains rely more on hospital-based transmission for survival, then nonspecific infection-control measures will disproportionately reduce R infections (17); in the simplest case they will have no impact on the equilibrium frequency of patients harboring S strains, but only reduce the frequency of harboring R strains (18). To the extent these models are to be believed, the no-infection counterfactual seems most relevant.
4. *Vaccination* must be considered in 2 different ways.
  a) *Vaccination against pathogens that can cause drug-resistant infections*. Many vaccines protect against all strains of an infection, regardless of drug resistance. To the extent that vaccination reduces absolute incidence of disease, and with it the absolute incidence of resistant disease, the no-infection counterfactual is relevant. Some complexity arises when the vaccine reduces incidence of some types of disease but increases incidence of others (e.g., serotype replacement in pneumococcal conjugate vaccines (19)). Furthermore, some vaccines that are selective for certain types, like pneumococcal conjugate vaccines, may disproportionately reduce drug-resistant disease (20), but this effect appears to be temporary, because resistant nonvaccine types increase in frequency (21). Notwithstanding these complexities, we believe the no-infection counterfactual is the most relevant here.
  b) *Vaccination against pathogens (e.g., influenza, respiratory syncytial virus) that are associated with antimicrobial use* (22). Here, the impact on AMR is a side benefit of reducing other infections that trigger antibiotic treatment, and we can see these vaccines as strategies to reduce antibiotic use (strategy 1), for which the S-infection counterfactual is relevant.
5. *Improving access to water, sanitation, and hygiene*. Here, as with vaccination or infection control, the primary effect is to reduce infection rates with all pathogens, regardless of resistance status. Thus, we would expect the no-infection counterfactual to be most relevant.
6. *Introduction of new antibiotics.* This effectively renders a previously R infection S, by expanding the options for treatment. This is the case where the S-infection counterfactual is the most clearly relevant.
7. *Reduction of environmental or agricultural antimicrobial use.* Although the role of agricultural antibiotic use and environmental antibiotic residues in promoting resistant human disease remains difficult to quantify (23)*,* the basic way in which such pressures likely act is to increase the proportion of resistant bacteria among those to which humans are eventually exposed. Reducing such use would thus seem likely to shift human exposure toward a more susceptible flora, making the S-infection counterfactual most relevant. However, a better understanding of mechanisms (24) might change this view.
8. *Livestock vaccination, infection control, food-animal handling changes, and other measures to reduce animal-to-human transmission of infection.* To the extent that such measures are used as part of a One Health strategy to reduce human exposure to foodborne pathogens, the no-infection counterfactual appears most relevant.
9. *Combinations of interventions.* Interventions to control AMR are very often deployed in combinations. A combination of interventions will have effects that depend on the details. In the case of “resistance-targeted infection control” recently modeled (13) and already in practice, to some extent (e.g., selective decontamination of carriers of resistant hospital-transmitted bacteria), this is an explicit effort to reduce the relative fitness of R strains, with the expectation of their replacement by S strains. Thus, the S-infection counterfactual is most relevant. In general, interventions may bundle approaches that will reduce total infections and approaches that will reduce R infections specifically, underlying the importance of estimating both counterfactuals.

### Role of competition

In most of the cases in the preceding list where the S-infection counterfactual was thought to be relevant, this conclusion depended on the assumption that R and S strains compete. If so, inhibiting the transmission of the R strain leads to an increase in the transmission and disease from the S strain. For most infections, there is limited evidence to address this question. Much of the existing evidence comes from analyses that have tried to distinguish between the hypothesis that R infections *add to* the total burden of infection (addition) versus the hypothesis that R infections *replace* S infections (replacement) (25, 26), with the 2 hypotheses pointing toward the no-infection and S-infection counterfactuals, respectively.

Here we review what is known for selected species.

- *Multiple pathogens*. From analysis of European surveillance data on 5 major bacterial pathogens, including the 3 specifically discussed here, researchers concluded that R infections mainly added to the burden of total infections rather than replacing S infections (27). Likewise, a cross-hospital comparison of bloodstream infection trends in 14 hospitals for methicillin-resistant *Staphylococcus aureus* (MRSA) versus methicillin-susceptible *S. aureus* (MSSA), as well as other antibiotic-resistant pathogens, found mainly evidence of addition rather than replacement (addition/no-infection) (26).
- *Vancomycin-resistant Enterococcus faecium.* Early work suggested that hospital-adapted clones of vancomycin resistant *E. faecium* were genetically distinct from susceptible members of the same species, carrying 100 hospital-specific genes (28, 29). More recently, vancomycin-resistant and vancomycin-susceptible *E. faecium* strains have been found phylogenetically intermingled, suggesting less niche adaptation in the latter case and more potential for competition (30). A longitudinal study in a hospital found no evidence that vancomycin susceptible *E. faecium* colonization was protective against acquisition of vancomycin resistant *E. faecium* (31), arguing against competition (unclear).
- *MRSA.* The same longitudinal study did find evidence that MSSA carriage reduced acquisition of MRSA in hospitalized patients (31), supporting a role for competition. However, a systematic review found that the increase of MRSA over the 2000s was mainly consistent with addition to, rather than replacement of, MSSA incidence (25). The same pattern has been seen in the more recent decline of MRSA. A study in 2 Boston hospitals showed this decline was accompanied by steady incidence of MSSA infections (32). (addition/no-infection).
- *Streptococcus pneumoniae.* Geographic correlations of resistance with antimicrobial use (33, 34) and phylogenetic evidence of frequent loss and gain of resistance within pneumococcal lineages (35) suggest the existence of competition between R and S lineages (replacement/S infection).
- *Escherichia coli*. Trend analysis of *E. coli* bloodstream infections in the Oxfordshire district, United Kingdom, from 1999 to 2011, showed a 30% increase in the overall incidence in 2006 and 2007. This coincided with a 2-fold increase of resistant cases, whereas incidence of susceptible cases remained stable during the study period (36), rendering competition less plausible (addition/no-infection).

### Scenarios in which the no-infection counterfactual would always apply 

Sometimes an infection occurs, or reaches a certain level of severity, only because the organism causing it was resistant to an antibiotic that was administered to the host. This may be the case, for example, when prophylaxis or preventive therapy fails because the organism is resistant to the antibiotic used (37). One apparent example of this phenomenon is increasing failure rates of antibiotic prophylaxis in colorectal surgery, associated with growing prevalence of resistance in Enterobacteriaceae (38). Another scenario involves the failure of treatment for mild infections, such as urinary tract infections, when they are resistant, leading to a higher proportion of cases that progress to more severe outcomes, such as septicemia. Results of a small individual-level study showed that multidrug-resistant (MDR) urinary tract infections are a risk factor for progression to sepsis (39). Ecological comparisons show a strong correlation between the prevalence of various resistance phenotypes in Enterobacteriaceae and *Pseudomonas* and the incidence of septicemia (40), consistent with such a scenario. Finally, a study of invasive *S. pneumoniae* disease showed that a particular allele of a penicillin-binding protein that reduces β-lactam susceptibility was associated with meningitis (vs. other forms of invasive disease) after adjusting for other predictors (41). The same positive association with meningitis was found for resistance to a number of other drugs, including non-β-lactams. The mechanism remains unclear. More work is needed to understand the extent of this phenomenon, but to the extent it is relevant, it suggests increased relevance of the no-infection counterfactual.

### Selecting a counterfactual

The foregoing suggest that which counterfactual is most relevant may depend on the intervention being envisioned: this is clearest for the case of a new antibiotic, whose potential impact is most clearly related to the question of how much worse the outcome of an R infection is than the outcome of an S infection (S-infection counterfactual). More subtly, it may be related to the intervention and the species, because different species show more or less evidence of competition or replacement between R and S strains, which is, in many cases, a precondition for the S-infection counterfactual to be relevant. In specific situations, only the no-infection counterfactual may be relevant, because antibiotic exposure would prevent any S infections.

Others have suggested, in a somewhat similar spirit, that the S-infection counterfactual is most relevant to an interest in the impact of inappropriate therapy, whereas the no-infection counterfactual is most relevant to understanding the public health impact of R infections. We agree with the first point but would argue that the intervention-centric perspective shows that both counterfactuals may be relevant from a public health perspective (42). Given these complexities, we advocate estimating the burden of AMR, where possible, using both counterfactuals and propose that the resulting numbers, which may be very different, will provide bounds on the true maximum impact of an intervention to control AMR. In the following sections, we describe how that might be done.

## DESIGNING STUDIES TO MEASURE BURDEN

There are different ways to determine the burden of a disease. For the global burden of disease studies conducted yearly by the Institute for Health Metrics and Evaluation, information about the underlying cause of death is extracted from death certificates, based on *International Statistical Classification of Diseases and Related Health Problems* coding. This has been applied for AMR as well; UK-based researchers examined death due to MRSA on the basis of it being mentioned on death certificates with *International Statistical Classification of Diseases and Related Health Problems* codes indicating staphylococcal infection as the underlying cause of death (43, 44). This method has not become widespread, because MDR pathogens are very rarely reported on death certificates (42, 45) and, if reported, may not be the true underlying cause of death (46). Other types of registries underperform as well; thus, purpose-designed studies are used to fill this void, among which cohort studies are considered the most reliable method to come to objective, attributable mortality estimates for infections caused by MDR pathogens (Table 1) (42, 47).

**Table 1:** Different Methods to Determine the Burden of Disease, Using Death as a Primary Outcome With the Benefits and Challenges for Application to the Domain of Antimicrobial Resistance

**Method**	**Pro**	**Con**
Registry-based methods		
Death certificates (ICD coding)	Available from national registry	Only lists underlying cause of death
Avoidable deaths	Registered EU vital statistic	Based on predefined list of conditions considered avoidable (does not include AMR)
Case-fatality rate	Objective	Requires registration of number of patients infected by resistant pathogens
		No distinction between dying with or because of an infection
		Deaths can be double counted for different causes
Purpose-designed studies		
Disease-related death	Based on individual patient data	Subjective
	Clinical judgment	Resource intensive
		External validity
Attributable death: cohort studies	Counterfactual approach	External validity
	Objective	Choice of control group
		Requires proper adjustment for confounders
		Primary outcome is odds ratio or hazard ratio

Abbreviations: AMR, antimicrobial resistance; EU, European Union; ICD, *International Statistical Classification of Diseases and Related Health Problems*.

Thus far, we have made the case that studies should estimate the burden of AMR by considering 2 counterfactual scenarios: the S-infection counterfactual and the no-infection counterfactual. To make such a comparison in a cohort study, 3 different cohorts need to be defined: patients with R infection, patients with S infection, and uninfected patients. Conceptually it is easy to distinguish among these cohorts, but translating these concepts into cohort-inclusion criteria and selection methods creates some challenges. Also, AMR burden is often measured as attributable mortality, or a derived outcome like disability-adjusted life-years. Though mortality seems like an objective outcome, it can be measured at different points in time, within differently defined populations. To ensure internal and external validity of burden estimates, the outcome definition should inform the applied analytical approaches, which, unfortunately, is often not the case (48).

### Who are patients with R infection, S infection, or no infection?

#### R or S infection.

In determining the burden of resistance, the underlying hypothesis is that infections with drug-resistant pathogens will have worse outcomes than those caused by drug-susceptible pathogens. There are a number of possible pathways for worse outcomes (in otherwise identical patients) if the resistance profile of the pathogen is such that first-line antibiotics are no longer effective: 1) time to appropriate therapy could be longer (49), 2) “reserve” antibiotics could be less effective (e.g., vancomycin) or more toxic (e.g., colistin) (50), 3) the infection could be untreatable due to patients’ lack of access to alternative antibiotics (51) or pan-drug resistance (52, 53), 4) prophylactic antibiotic treatment could have failed to clear the infection-causing pathogen (discussed in a later section) (38), and 5) resistant infections could be more virulent than susceptible ones (54). Based on these considerations, the problem of drug resistance could be translated as MDR, difficult-to-treat resistance, or untreatable resistance. From a treatment perspective, the problem of drug resistance could be defined as receiving inappropriate (prophylactic) therapy and, from a pathogen perspective, as increased virulence.

Most commonly, the impact of resistance has been established by assigning patients on the basis of indicators of MDR of the infectious agent (55): MRSA versus MSSA (56, 57), third-generation cephalosporin-resistant versus susceptible *E. coli* (56, 58), or carbapenem-resistant versus susceptible Gram-negative bacteria (59). Difficult-to-treat resistance has been applied as well, defined as Gram-negative pathogens nonsusceptible to all β-lactams including carbapenems and fluoroquinolones (60)—basically an extension of MDR. The caveat here is that none of these definitions is equal to inappropriate treatment, because a large proportion of the patients in these studies received second- or third-line antibiotics for empirical treatment, probably on the basis of clinical presentation of the patient and/or susceptibility patterns in past isolates in the same facility. For difficult-to-treat resistance, the public health relevance also seems limited, because incidence in the cited study was only 1%. Even though AMR is an increasing problem, the number of patients with bacterial infections that can be considered untreatable, especially within 1 setting, or for 1 specific group of pathogens, is still too limited to be applied as a definition.

Another approach involves the comparison of patients with appropriately treated infections versus those who did not receive appropriate treatment (61, 62). Conceptually this answers a different question from the impact of resistance versus susceptibility, though it is related. A key problem here is how to define appropriate therapy. Antibiotic resistance is not binary, but is expressed as the minimal inhibitory concentration for a particular drug. Translating from this continuous scale to a binary determination of resistant or susceptible (and thus of inappropriate or appropriate therapy) can depend on the pharmacodynamics and pharmacokinetics of the antibiotic in combination with the location of the infection, dosing, administration method, and patient characteristics (e.g., distribution volume, clearance). Most studies have a practical approach and define appropriate therapy on the basis of in vitro activity of the administered drug, which is only 1 part of the equation. Another important caveat is that antibiotic therapy can be adjusted over time, informed by clinical progress or laboratory results. If therapy over longer periods is considered to determine appropriateness, immortal time (63) or collider stratification (64) can bias results. Immortal time bias refers to a span of follow-up time, during which, because of the exposure definition (therapy adjusted after 24 hours), the outcome (death) could not occur. If this is neglected during the analysis phase, the preventive effect of therapy on death will be overestimated (63). Collider-stratification bias is a type of selection bias whereby a spurious association is created between exposure and outcome by conditioning on a common consequence of both (65); for example, conditioning on the APACHE II score on day of infection onset when assessing the association between empirical treatment and survival. At the same time, in observational studies, confounding by indication plays a role. Antibiotic treatment is assigned on the basis of patient characteristics, such that more severely ill patients have a higher chance of being treated with second-line, (more often) appropriate antibiotics, but these same patients are also more likely to have a worse outcome, resulting in an underestimate of the effect of inappropriate therapy (66, 67). Given these pitfalls, the validity of using appropriate treatment measures to determine the impact of resistance is debatable.

There have been laboratory studies showing that, for certain pathogens, drug resistance is associated with increased virulence, especially for *Acinetobacter baumannii* (68–70), *Pseudomonas aeruginosa* (71), *E. coli* (54), or *S. aureus* clones (72). However, worse clinical outcomes have not been confirmed in clinical studies focusing on specific virulent clones, like *E. coli* ST 131 (73), virulent *P. aeruginosa* strains (74), or Panton-Valentine leukocidin-positive MRSA (75).

On the basis of these considerations, the application of MDR profiles appears to be the most rational approach to establish the burden of resistance; it is an objective exposure, and clear definitions of MDR have been developed for different species (55). It also combines a higher probability of inappropriate therapy with the possible impact of virulence factors that could be associated with resistance. This exposure is also determined by data collected at the time of diagnosis of infection and, as such, is not influenced by clinical progress or other time-related factors. Future mediation or subgroup analysis could further disentangle the roles of inappropriate therapy and virulence and provide more insight in the true underlying processes of the additional burden of AMR.

Before we can continue the discussion, it is important to emphasize the distinction between hospital-onset and community-onset infections; these patients come from different at risk populations. For the former, the study base consists of hospitalized patients, whereas for the latter, it consists of members of the community. Members of the R, S, and no-infection cohorts should always be selected from the same study base.

#### No infection.

For the no-infection counterfactual, patients free of the infection under study must be selected. As opposed to the R and S cohorts, microbiological confirmation (i.e., a negative preexisting culture) should not be a prerequisite for the no-infection cohort. Rather, the criterion should be the absence of a positive culture, either because a sample was not collected or because 1 or more samples were collected and cultured but none was positive. The clinical decision to culture is an indication of the presence of certain risk factors and/or symptoms. As such, conditioning on a negative culture can create a form of selection bias (76), which should be avoided. Because most patients in the community or hospital, at a certain moment, will not be infected, the risk of misclassification bias by including patients in this cohort, when they lack a microbiological culture, is generally low, especially for serious diagnoses like bloodstream infections. Nevertheless, microbiological testing to confirm a participant’s eligibility for the no-infection cohort can be applied without risk of bias. This would be most important in resource-poor settings where diagnostic procedures will be underused, which will increase the risk of misclassification. In these settings, extra exclusion criteria could be applied as well, like antibiotic treatment in combination with a clinical diagnosis of infection, or active case finding can be funded and incentivized during the study.

Finally, it is important to consider whether other types of infections than the one of interest, or caused by pathogens other than the one under study, are allowed within the no-infection cohort. One should not condition on future events, as further explained later in this article; thus, this usually precludes exclusion of patients on the basis of other exposures than the one of interest.

### Selection criteria for patients within the different cohorts

#### Ratios of R to S and of R to no infection.

Generally, in cohort studies, all patients with R infections in a predefined population are included in the study; it is the exposure of interest and the number of cases that are usually limited. Including all R-infected patients maximizes study power and ensures representativeness. Because resistance proportions seldom exceed 50%, this means that, in the same predefined population, there will be more S infections. Because infections in the community or hospital are generally rare events, especially when focusing on a specific pathogen, there will be even more patients eligible for the no-infection cohort. The question is whether all these patients should be included for the comparison, or whether data collection can be more efficient.

If one is not solely interested in the burden of resistance but would like to estimate the burden of infections in general, or S infections specifically (as a secondary objective), all S infections could be selected using the same argument just discussed. However, if the main objective of the study is to determine the burden of resistance, it follows that the inclusion ratio of R to S, or R to no-infection, can be higher than the true ratio. Especially because, from ethical, privacy, and cost perspectives, it is recommended to restrict data collection to the minimum required for a statistically and clinically significant study. Some have suggested that ratios of up to 1:4 could result in noticeably more precise estimates, especially if this means that the sample size can be increased (77). On the other hand, ratios of more than 1:1 will limit the choice of statistical tests and could negatively influence the power to detect a difference (78). In practice, most studies adhere to 1:1 ratios (59), and this equal allocation is probably most prudent with respect to a combination of precision, power, and costs. This raises the issue of how to select this subset of patients for the S-infection, as well as no-infection, cohorts, which we address next.

#### Selecting subcohorts for S and no infections.

There are basically 2 options to select a subset of patients from a full cohort: randomly or on the basis of matching. The advantage of matching is that it can increase efficiency and enables control of factors that are important but difficult to precisely measure, like fitness or immune status. This can be achieved by matching on a proxy, which can represent these unquantifiable, confounding factors. A proxy for fitness and immune status among hospitalized patients is the amount of time spent in a certain hospital ward before acquiring an infection (hospital-onset infections); in the community, the age of a patient can be used as a proxy (community-onset infections). Ignoring timing of the infection will seriously bias study results. Matching for time to exposure, therefore, often has been applied in studies assessing the impact of drug-resistant infections, especially in the hospital setting (57, 58, 79–82). Patients with R infections will then be matched to uninfected patients who stayed in the hospital at least as long as the exposed patients until onset of their infection (frequency matching). If more patients are eligible, extra matching criteria can further focus the selection, or random selection methods can be applied within the subset. Although this reduces the bias of hospital exposure, it also entails conditioning on a future event: the matched patients will remain R-infection free until discharge. This will complicate the interpretation of the comparison, because it becomes conditional on a future event: your current risk of death is X times lower without an R infection, if you do not develop an R infection later during your hospital stay (83).

**Figure 2:**
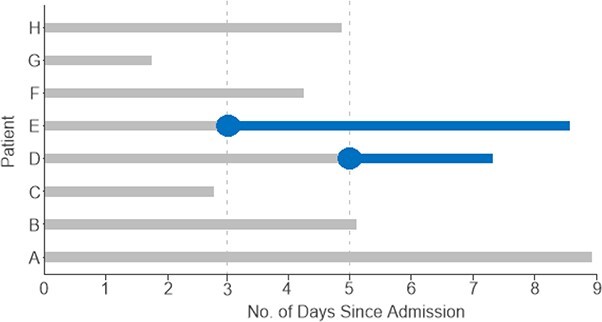
Illustration of exposure density sampling for matching patients from the resistant cohort and the no-infection cohort to study clinical outcome. Each horizontal line represents an admission, a blue circle represents a culture positive for a drug-resistant pathogen. Blue patient-days are attributed to the infection, gray patient-days are attributed to no infection. Patients D and E acquire a resistant, hospital-associated infection. On the basis of exposure density sampling, patient E can be matched at their time of positive culture to any patient staying >3 days and without infection on day 3: patients A, B, D, F, or H. Patient D is an appropriate match even though they become infected later. When patient D does become infected, patients A and B are the only appropriate no-infection matches for patient D.

A more advanced method to match for exposure time without conditioning on a future event is called exposure density sampling (84). Patients are still matched on the basis of time to R infection for the exposed patients, but the unexposed patients are allowed to become exposed patients after their enrolment (Figure 2). The infection will be considered a time-dependent exposure and, as such, we consider exposed and unexposed patient-days, as opposed to assigning a fixed exposure status to each patient for the entire study period. Wolkewitz et al. (84) have shown that this type of sampling results in unbiased estimates and provides better results than frequency matching.

Another method that is being developed for hospital epidemiology is the case-cohort approach. Here, all R infections are considered the cases. The cohort is a subcohort of all patients, including cases. This means that the patients in the subcohort will not need to be matched for exposure time until the onset of the infection among the cases. This will make selection of the subcohort logistically much more feasible. However, to come to unbiased estimates, data will need to be weighted, for which “skeleton” information of the full cohort is needed, like dates of admission and discharge, and life status for all patients in the selected hospital wards. It also performs best in settings with low infection rates and low censoring rates. This approach provides valid estimates when compared with a full cohort approach, but estimated confidence intervals can be larger (85, 86).

### Clinical outcomes and appropriate analytical approaches

The impact of drug-resistance is generally measured by morbidity or mortality outcomes, with a focus on morbidity for infections outside the hospital, and a focus on length of stay and mortality outcomes for infections among hospitalized patients. Length of hospital stay is a very objective and easily attainable outcome measure, and an important proxy for excess costs; as such, it is included in most studies looking at the burden of AMR. An obvious limitation of this outcome measure is that rapid death, the worst possible outcome, is associated with shorter length of stay. Death is another objective outcome measure, often measured at 28 days (based on sepsis criteria) or 30 days (monthly registration) after infection onset, or at hospital discharge (in-hospital death). Although the former 2 suggest that life status is determined for all patients at a certain point in time, it is often not specified whether 28-day or 30-day mortality data are actively collected and thus includes death among patients who are discharged alive. Studies should clearly report this because it determines the correct analytical approach.

In the case of community-onset infections, estimation of excess length of stay is straightforward, because the entire hospital admission period can be considered attributable to the infection. For hospital-onset infections, time from admission to infection needs to be subtracted, or acknowledged through matching as previously explained. Unfortunately, timing of infection is often ignored when calculating the excess length of stay of drug-resistant, hospital-onset infections, thus inflating its impact (87, 88). To avoid time-dependent bias, multistate models (89) can be applied. In multistate models, several possible events can be included, like hospital admission, infection, discharge, and death. Individuals move into these different states, whereby the assigned infection status of individuals changes over time, acknowledging the time dependency. Transition hazards between states can then be used to calculate the average excess length of stay for infected patients (90). Inverse probability-weighted Kaplan-Meier models (91) have been suggested as an analytical approach as well; these have the added advantage of being able to include time-dependent confounders.

Because infection in hospital is a time-dependent exposure, survival models should be applied to analyze the difference in mortality risk. Survival models censor all patients lost to follow-up, which assumes that these patients have the same risk of dying as patients still in the risk set. If all patients are followed up for a certain period after infection, independent of hospital discharge, this assumption holds. However, if follow-up ends at hospital discharge (i.e.,in-hospital death is being measured), patients who are discharged alive have zero chance of experiencing the outcome; discharge alive and in-hospital death are competing events. In this case, patients lost to follow-up (discharged alive) should not be considered censored, because this would overestimate the impact of the time-dependent exposure on hospital death. Different types of survival models can be applied that acknowledge competing events, including the subdistribution (Fine and Gray) approach, the cause-specific approach (92), parametric mixture models (93), the Aalen additive hazard models, or marginal structural models with inverse probability weighting (94). As in all observational studies, important confounders, like severity of illness, presence of comorbidities, and age, should be incorporated in the analysis, either through stratification, multivariate modeling, or inverse probability weighting.

## CONCLUSIONS

In this article, we have provided a framework for studies that aim to establish the burden of AMR. We addressed the conceptual challenge—“compared to what?”—through an intervention-based causal approach. The S-infection and the no-infection counterfactual were introduced, as were their link with intervention strategy, the considered species or type of infection, and prophylaxis versus treatment. Preventive interventions (e.g., vaccines, improved sanitation) generally reduce the frequency of drug-resistant and drug-susceptible infections equally, pointing toward the no-infection counterfactual. Improved antibiotic treatment reduces selective pressure, causing replacement of R infections by S infections, pointing toward the S-infection counterfactual. This is true if competition exists between R and S strains, which, so far, has been shown convincingly only in a few cases. In specific cases, infections only occur because the causative pathogen was resistant to the administered antibiotic (e.g., surgical site infections after prophylaxis); consequently, the no-infection counterfactual should be applied. For assessing the value of new drugs, the S-infection counterfactual is directly relevant. Given these complexities, we advocate estimating the burden of AMR using both counterfactuals. The resulting numbers will provide policy-relevant information about the upper and lower bound of future interventions designed to control AMR.

The second question that we discussed is how to establish the burden. National registries of death certificates or other nonspecific registries underperform and, as such, purpose-designed cohort studies are currently the most reliable method. R-, S-, and no-infection cohorts should be selected, whereby a subset of all patients in the S- and no-infection cohort can be selected through exposure density sampling, acknowledging the importance of time to infection and preventing conditioning on future events. Agreed definitions of MDR profiles should be used to distinguish between R and S infections, combining a higher probability of inappropriate therapy with the possible impact of virulence factors. Finally, data should be analyzed by time-to-event methods, acknowledging the time dependency of the infection and important confounders. Competing events should be considered if patients are not followed up beyond hospital discharge.

Different initiatives are underway to determine the global, regional, or national burden of AMR. Hopefully, this framework will help harmonize and improve approaches, resulting in reliable, actionable data that will halt the increase of AMR and preserve antibiotics for future generations.
